# The effects of numeracy and presentation format on judgments of contingency

**DOI:** 10.3758/s13421-020-01084-8

**Published:** 2020-08-26

**Authors:** Susan Cooper, Frédéric Vallée-Tourangeau

**Affiliations:** Department of Psychology, Kingston University, Kingston-upon-Thames, KT1 2EE UK

**Keywords:** Causal judgment, Numeracy, Representational effects

## Abstract

Covariation information can be used to infer whether a causal link plausibly exists between two dichotomous variables, and such judgments of contingency are central to many critical and everyday decisions. However, individuals do not always interpret and integrate covariation information effectively, an issue that may be compounded by limited numeracy skills, and they often resort to the use of heuristics, which can result in inaccurate judgments. This experiment investigated whether presenting covariation information in a composite bar chart increased accuracy of contingency judgments, and whether it can mitigate errors driven by low numeracy skills. Participants completed an online questionnaire, which consisted of an 11-item numeracy scale and three covariation problems that varied in level of difficulty, involving a fictitious fertilizer and its impact on whether a plant bloomed or not. Half received summary covariation information in a composite bar chart, and half in a 2 × 2 matrix that summarized event frequencies. Viewing the composite bar charts increased accuracy of individuals both high and low in numeracy, regardless of problem difficulty, resulted in more consistent judgments that were closer to the normatively correct value, and increased the likelihood of detecting the correct direction of association. Findings are consistent with prior work, suggesting that composite bar charts are an effective way to improve covariation judgment and have potential for use in the domain of health risk communication.

**Electronic supplementary material:**

The online version of this article (10.3758/s13421-020-01084-8) contains supplementary material, which is available to authorized users.

## Introduction

The covariation or contingency between two events concerns the degree to which they are associated and may be defined in terms of their co-occurrence—that is, the extent to which one event is likely to occur given the presence or absence of the other event. In the absence of expertise or prior beliefs on which to base a conclusion, the evaluation of contingency information can be used to infer or refute a potential causal relationship and obtain an estimate of its strength. Therefore, judgments of contingency are often central to everyday human behaviour and decision-making. For example, a student might be interested in whether a revision program boosts examination success before deciding whether to enroll, and a patient with a skin condition might wish to ascertain whether a treatment is effective before deciding whether to use it or not. Given that such judgments can have far-reaching consequences, it is important to understand how they are arrived at and the factors that influence them, including how best to present contingency information to aid comprehension and facilitate optimal decisions.

**Fig. 1:**
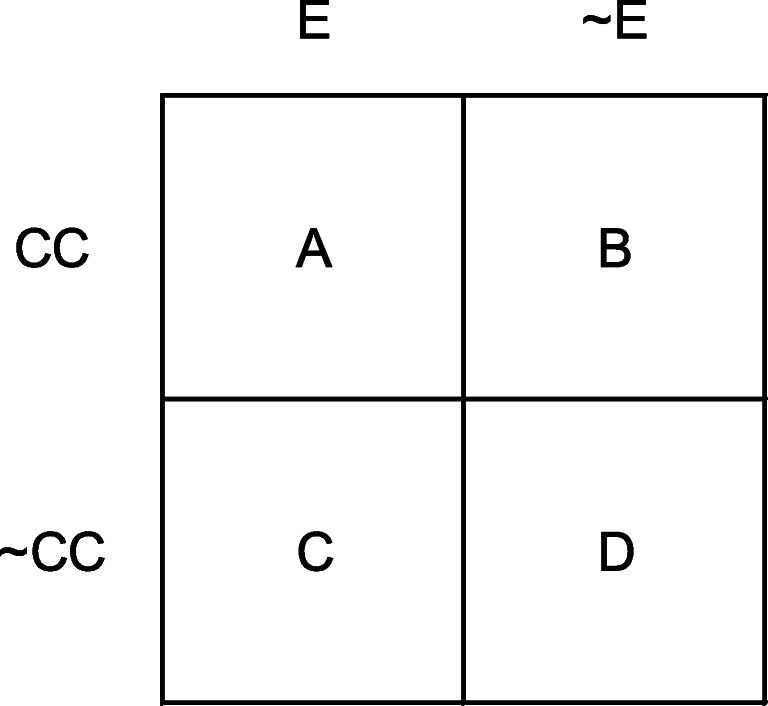
A 2 × 2 contingency table. CC = candidate cause present; ~CC = candidate cause absent; E = effect present; ~E = effect absent. A, B, C, and D represent individual cell frequencies

When considering whether one event (the *candidate cause,* or CC) influences the occurrence of a second (the target effect), there are four categories of information, and this may be given in a 2 × 2 contingency table (as shown in Fig. 1), where the rows refer to the presence and absence of the CC, and the columns refer to the presence and absence of the effect. Conventionally, Cell A contains the frequency with which the CC and the effect co-occur, Cell B the frequency that the CC is present and the effect does not occur, Cell C the frequency that the effect is present in the absence of the CC, and Cell D contains the frequency with which the CC and the effect are both absent. Thus, Cells A and D contain confirmatory evidence of a causal relationship, and Cells B and C contain disconfirmatory evidence.

A normative approach to evaluating the strength and direction of a relationship from contingency information is to calculate the difference between two conditional probabilities: the probability of the effect given the CC and that of the effect in the absence of the CC (Ward & Jenkins, 1965), or ΔP (Allan, 1980; Kao & Wasserman, 1993):

1 $$ \Delta \mathrm{P}=\mathrm{P}\left(\mathrm{E}|\mathrm{CC}\right)\kern.5em -\kern.5em \mathrm{P}\left(\mathrm{E}|\kern.3em \sim \kern.3em \mathrm{CC}\right)=\frac{A}{A+B}\kern.5em -\kern.5em \frac{C}{C+D}. $$

While this normative metric weighs cell frequencies equally, a long history of studies has demonstrated that when making a covariation judgment there is a nonnormative tendency to consider evidence supporting a positive association to be more relevant; in particular, the order of cell importance is perceived to be A > B > C > D (e.g., Crocker, 1982; Shaklee & Tucker, 1980; Smedslund, 1963; Wasserman, Dorner, & Kao, 1990). This differential weighting explains several alternative strategies, or heuristics, which vary in terms of complexity and sophistication (Arkes & Harkness, 1983; Shaklee & Tucker, 1980; Ward & Jenkins, 1965).

Least sophisticated is the Cell A heuristic, in which only the contents of Cell A are considered: A positive covariation is inferred if the frequency of these co-occurring incidences is greater than all other cell frequencies, and a negative covariation is inferred if it is smaller. This has also been termed a *positive hits* strategy (McKenzie, 1994), and observed by many researchers (Arkes & Harkness, 1983; Shaklee & Tucker, 1980; Ward & Jenkins, 1965). Alternatively, a *positive test* strategy (Klayman & Ha, 1987) relates to a tendency to focus on cases that contain the property of interest, and therefore involves an examination of Cells A and B (the probability of the effect given the cause) and/or Cells A and C (the probability of the cause given the effect). Similarly, a *proportion of hits* (McKenzie, 1994) or *A versus B* (Shaklee & Mims, 1981) strategy deems these two cells most pertinent, and a relational judgement is made by using the frequencies therein to calculate the probability of the effect given the cause. Another strategy which considers the contents of Cells A and B requires simply to calculate their difference (i.e., A − B)—a *hits minus false positives* strategy (McKenzie, 1994, supported also by Arkes & Harkness, 1983). These nonnormative strategies (Cell-A/positive-hits, positive testing, proportion-of-hits/A-versus-B, hits minus false positives) all have in common that they only consider the contents of one or two cells, the others (notably Cell D in all instances) are disregarded. More demanding is the *sum-of-diagonals* heuristic (Arkes & Harkness, 1983; Shaklee & Mims, 1981; Shaklee & Tucker, 1980), in which confirmatory evidence (the sum of Cells A and D) is compared with disconfirmatory evidence (the sum of Cells B and C); a positive association is assumed if (A + D) > (B + C), and a negative association if (A + D) < (B + C). It is important to note that although both the sum-of-diagonals and the ΔP strategies make use of all four cell frequencies, only ΔP is normative and will provide an accurate judgment for all problems. Furthermore, because different strategies can produce the same judgment for some problems, a sophisticated strategy cannot be inferred from accuracy alone (Allan, 1980; Shaklee & Wasserman, 1986).

Although many studies have demonstrated this cell weight inequality, and the consequential use of the various strategies described, few have tried to explain why the inequality occurs in the first place. It is important to understand the underlying mechanisms for this effect so that boundary conditions can be anticipated and, if necessary, appropriate interventions put in place. One explanation is that people assess evidence in a way that places greater weight on confirming a hypothesis than on disconfirming it (e.g., Wason, 1960), and therefore tend to test a hypothesis by examining cases that are known to have the property of interest rather than those that lack it (Klayman & Ha, 1987). Crocker (1982) also suggests that individuals regard positive confirming cases as most relevant when forming a judgment, and negative confirming cases least relevant. Thus, when considering covariation information, Cell A would be considered most salient as it contains the frequency of positive confirming cases which concern both properties of interest (the candidate cause and the effect), Cells B and C less influential as they each concern only one such property. In turn, Cell D would be considered least important as neither property is present in the negative confirming cases represented by this frequency. Cell B will receive somewhat more emphasis than Cell C since a comparison of Cells A and B (the probability of the effect given the cause) is more consistent with a temporal order of causation than a comparison of Cells A and C (the probability of the cause given the effect). There is often also a greater attention to the sufficiency of rules than to their necessity—in other words, it is less surprising that the effect might occur without the candidate cause, than that the effect might not occur when the candidate cause is present.

This tendency to focus on the target class in judgments of probability can be termed *denominator neglect*, whereby too much attention is given to numerators (the number of times an event happens) and insufficient attention to the overall number of opportunities for the event to happen, which is the denominator in the calculation of probability (Reyna & Brainerd, 2008). Thus, when presented with covariation information, the focus would be on the magnitude of the frequency in Cell A since it is the numerator of both P(E|CC) and P(CC|E), followed by Cells B and C as the numerators of P(CC|~E) and P(E|~CC) respectively, to the neglect of Cell D, which is only incorporated into denominators. Denominator neglect can be accounted for by fuzzy-trace theory (FTT; Reyna, 2004; Reyna & Brainerd, 1994), a dual-process model of memory and reasoning in which not only verbatim but gist (less precise, hence “fuzzy”) representations of relevant frequencies are encoded, and confusion occurs because the target class is included in the denominator that encompasses both target and nontarget classes. This theory suggests a preference for reasoning with the most essential, gist-based representations permissible for a given task—which is often sufficient to deliver an accurate solution—whenever possible. Hence, when comparing probabilities, the salient gist of the problem is the relative size of target categories (the numerators); their comparison therefore becomes the basis for a judgment, and the application of any cued knowledge about ratios is stymied because of confusion created by the overlap between the size of these target categories and that of the overall classes from which they were drawn (the denominators).

### Numeracy

Numerical information must frequently be considered when making decisions in a wide variety of scenarios (e.g., nutritional information on food labels, prices for best value, and risk information for insurance and health choices). However, the evaluation of numbers can be difficult because they can appear in different forms (e.g., 5%, 1 in 20, 0.05), and some manipulation or integration may be required (e.g., 5% off, buy two get one free). Furthermore, the same value may warrant a different interpretation depending on the context in which it is presented (e.g., a 10% pay rise might be considered good, but a 10% risk of disease as bad). Numeracy, or the ability to process probabilistic and numerical concepts, is therefore often required to make good choices (Peters et al., 2006; Reyna & Brainerd, 2008). Research suggests that numeracy levels vary substantially across the population. In particular, the association between numeracy and the understanding of risk information appears to be a very robust phenomenon (for reviews, see Peters, Hibbard, Slovic, & Dieckmann, 2007; Reyna, Nelson, Han, & Dieckmann, 2009), numeracy accounting for unique variance beyond that accounted for by education and general intelligence (Peters et al., 2006), such that even highly educated individuals find it difficult to comprehend numerical information when making decisions (Lipkus, Samsa, & Rimer, 2001).

Research also suggests that numeracy goes beyond the ability to comprehend and transform numbers. For example, a higher level of numeracy has been associated with reduced susceptibility to context and framing effects (Peters et al., 2006), and, when compared with other cognitive factors such as working memory span and impulsivity, predicts normatively superior decisions owing to an association with a more conscious, thorough, and elaborative search for a solution. For example, Cokely and Kelley (2009) found that while the proportion of choices consistent with expected value in a series of risky choice problems (e.g., $125 or 30% chance to win $900) was significantly related to numeracy, protocol analysis revealed little evidence of expected-value calculations, but rather an elaborative heuristic search through a number of simple (albeit often number related) considerations (e.g., $900 is a lot more than $125, 30% chance to win is the same as 70% chance to lose). Alternatively, focus was on the gist of the problem (FTT; Reyna, 2004), and while all individuals may prefer to process surface features of a problem in this way, the highly numerate are more likely to derive a richer gist when the information is numeric. Thus, greater numeracy involves a reliance on superior number-related intuition supplemented with the conscious ability to recognize when an analytical mode (e.g., number integration) needs to be employed (Peters, 2012).

There has been much research regarding the role of numeracy in Bayesian reasoning, where it has been found to be positively related to accuracy (Chapman & Liu, 2009; Sirota & Juanchich, 2011; G. Vallée-Tourangeau, Abadie, & Vallée-Tourangeau, 2015; Wu, Meder, Filimon, & Nelson, 2017). Numeracy has also been investigated in health-related decision-making (Galesic, Garcia-Retamero, & Gigerenzer, 2009; Peters, Hibbard, et al., 2007; Reyna et al., 2009; Schwartz, Woloshin, Black, & Welch, 1997). The numerical reasoning required in this domain involves the ratio concepts (fractions, percentages, and proportions) that are fundamental to the understanding of risk and probability, and which are also particularly challenging and prone to biases that undermine judgment and decision-making (Reyna & Brainerd, 2007), notably denominator neglect, and ratio bias, which is the perception that a low-probability event is more likely when drawn from a larger sample, even when the probabilities are identical or slightly worse (e.g., 10/100 and 9/100 preferable to 1/10; Reyna & Brainerd, 2008). These studies demonstrate that higher numeracy is associated with better comprehension and integration of numerical information, leading to more informed decisions and better medical outcomes. For example, Schwartz et al. (1997) found that high-numeracy individuals were better able to use information about breast cancer reduction associated with mammography than those who were low in numeracy; Peters, Dieckmann, Dixon, Hibbard, and Mertz (2007) found the highly numerate chose better quality hospitals based on numerical information about medical outcomes than those who were lower in numeracy (for further examples, see Reyna & Brainerd, 2007). Clearly, poor numeracy might be a barrier to making good health choices. Therefore, careful consideration should be given to the presentation of numerical information, and how this interacts with numerical ability, to improve comprehension, aid evaluation, and encourage best decisions.

### Representational effects

Research into problem solving and judgment under uncertainty has demonstrated that the format in which information is presented to reasoners can have a dramatic effect on problem difficulty, and influence the cognitive strategies employed. In particular, graphical displays can facilitate the communication and comprehension of information by attracting and holding attention to a concrete, visual display, summarizing large amounts of data, and drawing attention to patterns (Lipkus, 2007). Furthermore, graphical representations can support mathematical problem solving as related information is grouped together, which reduces search time, cues the relevant comparisons, and assists computation (Larkin & Simon, 1987). Pertinent to the examination of causal inferences, it was found that judgments were more normative when covariation information was presented graphically in a cumulative frequency tree rather than as a set of four simple propositions (F. Vallée-Tourangeau, Payton, & Murphy, 2008). In short, a well-designed visual display can aid comprehension and evaluation by scaffolding mental computation with direct visual inferences.

One area in which the effect of presentation format has been much studied is that of health-risk communication. Research has documented that patients and health professionals alike struggle to grasp numerical concepts that are prerequisites for the accurate evaluation, comprehension, and communication of health-relevant risk information (Gigerenzer, Gaissmaier, Kurz-Milcke, Schwartz, & Woloshin, 2007). Decision-making aids such as visual displays have helped overcome some of these difficulties for individuals of varying cognitive ability, age, and background (for reviews, see Garcia-Retamero & Cokely, 2013, 2017). In particular, and replicating the effects of earlier FTT experiments, the use of icon arrays has been shown to enhance comprehension of health-related risks (Fagerlin, Wang, & Ubel, 2005; Zikmund-Fisher et al., 2008) and benefit low-numeracy individuals especially (Galesic et al., 2009). An icon array depicts a risk using a matrix of individual symbols, such as asterisks or stick figures, some of which are highlighted as being affected in some way. Thus, an icon array illustrates a part-to-whole relationship, and proportions are visually available to judge. However, although the addition of icon arrays to numerical information has been shown to reduce denominator neglect when comparing unequally sized treatment groups (Garcia-Retamero, Galesic, & Gigerenzer, 2010), the perceived seriousness of otherwise equivalent risks in two icon arrays was found to be greater when the array contained a larger overall number of icons (e.g., 1,000 rather than 100), which does fit with a ratio-bias effect (Galesic et al., 2009). Furthermore, Galesic et al. (2009) found that risks appear less serious when presented in an icon array relative to a numerical representation, suggesting this is because attention is drawn to what is often a large number of unaffected people. Hence, icon arrays may be used to emphasize either the number affected (numerator) or the total number at risk (denominator).

The importance of portraying the part-to-whole relationship in a graphical display to aid decisions involving risk was explored in a series of studies by Stone and colleagues (Schirillo & Stone, 2005; Stone et al., 2003). They refer to the classes of information in a risk ratio as “foreground” (target group, numerator), and “background” (total number at risk, denominator), and suggest that by drawing attention to one or the other it is possible to make a risk seem either large or small. In an earlier study, Stone, Yates, and Parker (1997) found that a graphical format that highlighted the number of people harmed (i.e., foreground information) by a negative event that occurred while using a certain product increased willingness to pay (relative to a numerical format) for an improved, safer product that reduced the risk of the event by 50%, even though the risk itself was extremely small (e.g., 30 in 5,000 vs. 15 in 5,000). However, Stone et al. (2003) demonstrated that when the graphical format displayed both the number harmed and the total number at risk (i.e., both foreground and background information), this effect was eliminated. They propose that the observed increase in professed risk-avoidant behaviour when foreground information is highlighted is caused by the perception that risk reduction is greater, which is in turn due to a focus on the difference in numbers harmed (e.g., 30 vs. 15) made salient by the graphical display, whereas when both foreground and background information are displayed either graphically or numerically, the focus is on the total at risk (e.g., 5,000), which makes the reduction appear small since it shows the chances of harm are slight with either product. Thus, when selecting a graphical display format, consideration should be given to its overall purpose—for example, whether the intention is to enhance quantitative understanding or to promote behaviour change.

A normative contingency judgment involves the comparison of two conditional probabilities which enumerate the proportion of cases where the effect occurs as a subset of those with, and without, the candidate cause. However, as predicted by FTT, conditional probabilities involving subsets can be particularly difficult to determine as the nested relationship can foster confusion regarding denominators (Wolfe, Fisher, Reyna, & Hu, 2012). Hence, it is important that presentation formats depicting covariation information draw attention to this relationship. Indeed, research has demonstrated that interventions which accentuate the appropriate denominators in problems involving nested subsets (e.g., Euler diagrams, icon arrays) result in more accurate assessments of conditional probability (Galesic et al., 2009; Garcia-Retamero et al., 2010; Wolfe, Fisher, & Reyna, 2013; Wolfe et al., 2012). As proposed by Stone et al. (2003), an alternative graphical format which overcomes these problems is the stacked, or composite, bar graph. Research into health risk communication also suggests that visual formats such as composite bar graphs promote accurate judgments of a part-to-whole relationship since the numerator (number affected) and the denominator (total under study) are represented proportionally (for reviews, see Ancker, Senathirajah, Kukafka, & Starren, 2006; Lipkus, 2007).

### The present experiment

Much of the research on the communication of risk has examined situations where the event probability is small. For example, in the health domain, most hazards (e.g., suffering from the side effects of a drug, developing breast cancer) have extremely low probabilities of occurring from an individual perspective. Therefore, communication techniques have focussed on low-probability risk magnitudes. Furthermore, and once again particularly in the health domain, studies have often investigated graphical representations that encourage the modification of risk-relevant behaviour, and hence draw attention to the risk even when it is small. Such representations do not necessarily enhance comprehension of the risk information in any absolute sense (e.g., Stone et al., 1997). However, in the case of contingency judgments, the probabilities involved may be of any level, and the focus is not only on a comparison to determine which of two options is preferable, but also to directly evaluate the *strength* of any preference, giving due consideration to all information provided. As such, this experiment adds to prior research into the effectiveness of graphical formats by assessing their potential to facilitate an enhanced and objective assessment of comparative risks.

The purpose of this experiment was twofold. The first was to investigate the impact of problem difficulty on causal judgments when contingency information is presented in a graphical as opposed to a 2 × 2 matrix form. A problem might be classified as easy if the employment of multiple strategies, including the very simplest, or commonly used heuristics (e.g., Cell A, A versus B, sum-of-diagonals) will result in a correct judgment. Although it would be impossible to infer which strategy was used, a correct response is more likely for such a problem simply because many strategies will achieve it. A difficult problem might be classified as one for which only a normative strategy (i.e., ΔP) will result in a correct judgment, and therefore one where the significant manipulation of cell frequencies (and numeracy) is required. There are of course other potential combinations of cell frequencies in a covariation problem, such that although the simplest strategies (e.g., Cell A) will be unsuccessful, some basic numerical manipulation (e.g., the comparison of confirmatory evidence in Cells A and D with disconfirmatory evidence in Cells B and C) will result in a correct judgment without having to calculate ΔP, and these might be classified as being of medium difficulty.

The second purpose of this experiment was to investigate the degree to which numeracy moderated the influence of problem difficulty on causal judgments in each presentation format. We conjectured that presenting the contingency information in a composite bar graph should lead to more accurate judgments overall relative to a contingency table (2 × 2 matrix) format and could scaffold judgment even for more difficult problems. Furthermore, numeracy should moderate the impact of problem difficulty when information is displayed in a contingency table, but to a lesser extent when displayed in a composite bar graph.

## Method

### Participants

One hundred and forty-nine individuals in total participated in this experiment. Twenty-two participants did not respond to all items in the questionnaire, so their data were excluded, leaving 127 complete responses in the analysis.^1^ The majority (92.9%) were recruited via social media (Facebook and Twitter), and the remainder were undergraduate psychology students who participated in exchange for course credits. Participants were aged 19–75 years (*M* = 42.3, *SD* = 14.2); 71.7% female; 22.1% reported no degree, 28.3% a bachelor’s degree, and 49.6% a postgraduate degree. Of the graduates and postgraduates, 24.2% had studied STEM subjects.

### Materials

The experiment was presented on Qualtrics. Individual differences were measured using a series of demographic questions (age, gender, and level of education), and the 11-item numeracy scale developed by Lipkus et al. (2001). This scale assesses practical knowledge of probabilistic concepts and simple mathematical operations, including the ability to comprehend and transform risk magnitudes given as percentages, fractions, decimals, and proportions. It is a widely used and accepted measurement instrument to assess numeracy in health and medical decision-making, as well as other judgment and decision tasks (e.g., Galesic et al., 2009; Peters et al., 2006), and for diverse populations (see Garcia-Retamero & Cokely, 2017, for further examples).

For the contingency judgment task, a fictitious scenario was presented describing an experimental plant fertilizer and tests to determine whether it promoted a certain exotic plant (the Lanyu) to bloom (adapted from Kao & Wasserman, 1993). The scenario was followed by three sets of covariation information concerning the number of plants that had or had not been given the fertilizer, and the number that had or had not bloomed. All participants were given this information as a set of four simple propositions, followed by a summarized version in either a contingency table or composite bar graph.

The problem for the participants was to determine whether each of the three fertilizers was effective in promoting the Lanyu to bloom. They were asked to indicate this on a scale ranging from −10 (the fertilizer has a strong negative effect on the plant’s blooming), through 0 (no effect), to +10 (the fertilizer has a strong positive effect on the plant’s blooming). Crucially, in all three cases the fertilizer had a slight negative effect on whether the Lanyu bloomed with ΔP = −.1, and hence the normatively correct response on the scale used in this experiment was −1.

### Design and procedure

The experiment employed a 2 × 3 mixed design. The between-subjects variable was presentation format: In the contingency table (CT) condition, the results for each fertilizer were given in the classic 2 × 2 matrix format; in the bar graph (BG) condition, the results were displayed in a horizontal composite bar chart. The within-subjects variable was problem difficulty: hard, medium, and easy.

**Fig. 2:**
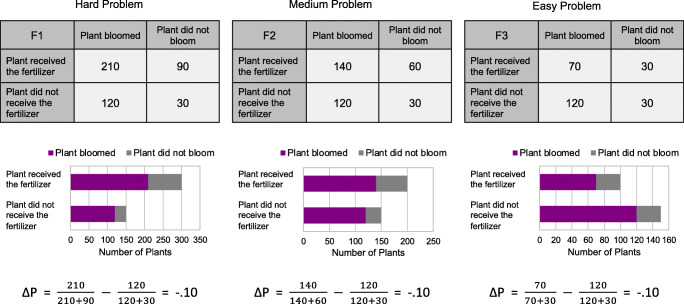
Contingency judgment problems as presented to participants in the contingency table and bar graph conditions for the hard (left panel), medium (middle panel), and easy (right panel) problems

To examine where a potential facilitating effect of presentation format occurs, the three problems were hierarchically structured to allow discrimination between possible judgment strategies. Thus, the results for the first fertilizer (F1, hard) were devised such that a correct contingency judgment could only be arrived at using ΔP, with Cell A containing the largest frequency, and (A + D) > (B + C) so that all nonnormative strategies (Cell A, positive testing, A versus B, hits minus false positives, sum-of-diagonals) would be unsuccessful in gauging the negative contingency. A correct judgment for the second fertilizer (F2, medium) could be obtained using either ΔP or the sum-of-diagonals heuristic; Cell A still contains the largest frequency so that strategies involving only Cells A and B and/or Cells A and C would be unsuccessful, but now (A + D) < (B + C), and hence might be evaluated accurately with a sum-of-diagonals strategy. Finally, for the third problem (F3, easy), a correct inference could additionally be based on the simplest Cell A strategy since A < C as well as (A + D) < (B + C). All three problems and their presentation in the two format conditions are shown in Fig. 2.

Participants first answered the demographic questions. Half were then randomly given the 11-item numeracy scale followed by the contingency judgment problems; for the other half, this order was reversed. Independent of this, participants were randomly assigned to either the CT or BG condition. The order in which the three covariation problems were presented was also counterbalanced across participants.

## Results

**Fig. 3:**
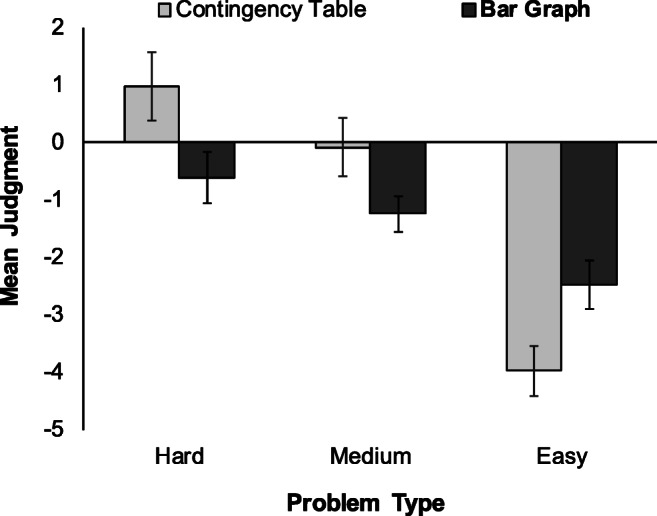
Mean judgment for the hard, medium, and easy problem in the contingency table and bar graph conditions. Error bars are standard errors of the mean

Of primary interest was accuracy in the contingency judgment task as a function of presentation format and level of difficulty. Figure 3 shows the mean judgment for each of the three levels of difficulty in each of the two presentation formats. In both the CT and BG conditions, judgments of contingency were more negative when problems were easier; however, this trend was more pronounced in the CT condition, judgments in the BG condition appearing more consistent across the three problems.

A 2 (presentation format, between subjects) × 3 (level of difficulty, within subjects) mixed analysis of variance (ANOVA) with a Greenhouse–Geisser correction^2^ revealed a significant main effect for level of difficulty, *F*(1.81, 225.95) = 39.18, *p* < .001, η_p_^2^ = .239, but no main effect for presentation format (*F* < 1). However, the interaction between presentation format and level of difficulty, *F*(1.81, 225.95) = 8.76, *p* < .001, η_p_^2^ = .065, was significant. As expected, simple effects revealed that for the hard problem, judgments of contingency were significantly more accurate in the BG condition (*M* = −0.61, *SD* = 3.66) than in the CT condition (*M* = 0.98, *SD* = 4.80), *t*(115.89) = 2.09, *p* = .038, Cohen’s *d* = 0.37, 95% CI [0.02, 0.72]. Similarly, for the medium level of difficulty, judgments were closer to the normatively correct value of −1 in the BG condition (*M* = −1.24, *SD* = 2.54) than in the CT condition (*M* = −0.08, *SD* = 4.02), although this difference failed to reach significance, *t*(104.50) = 1.95, *p* = .054, *d* = 0.35, 95% CI [−0.01, 0.70]. There was also a significant difference in judgments for the easy problem, *t*(125) = −2.48, *p* = .014, *d* = −0.44, 95% CI [−0.79, −0.09], once again responses in the BG condition (*M* = −2.47, *SD* = 3.34) being more accurate than those in the CT condition (*M* = −3.97, *SD* = 3.47). These comparisons suggest that although the main effect of presentation format was nonsignificant, contingency was judged differently in the two conditions, but differences depended on the level of difficulty.

### The effects of numeracy on judgments of contingency

**Fig. 4:**
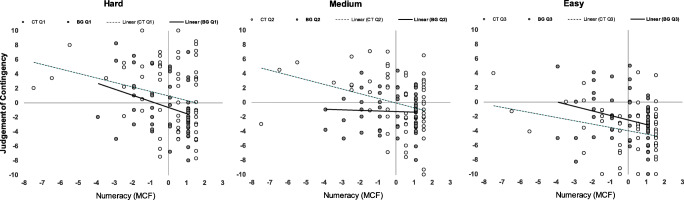
Contingency judgment as a function of numeracy (mean centred form) in the contingency table (CT; open circles) and bar graph (BG; dark circles) conditions for the hard (left panel), medium (middle panel), and easy (right panel) problems

The mean numeracy score for all participants was 9.67 (*SD* = 1.72) with 44.9% responding correctly to all 11 items: The distribution was highly skewed. Participants’ numeracy did not differ between the BG condition (*M* = 9.89, *SD* = 1.43, scores in range 6–11) and the CT condition (*M* = 9.44, *SD* = 1.96, scores in range 2–11), *t*(125) = −1.47, *p* = .144, *d* = −0.26, 95% CI [−0.61, 0.09]. Figure 4 plots judgment of contingency as a function of numeracy (mean centred form) in the two format conditions for each problem type. Regression slopes were negative, indicating that higher levels of numeracy are associated with lower or negative judgments. It can also be seen that gradients for the three problems are similar in the CT condition, but more variable in the BG condition. Correlations were significantly negative for all problems in the CT condition: hard, *r*(61) = −.255, *p* = .043, medium, *r*(61) = −.317, *p* = .011, easy, *r*(61) = −.261, *p* = .039. Correlations in the BG condition were also significantly negative for the hard, *r*(62) = −.327, *p* = .008, and the easy problem, *r*(62) = −.270, *p* = .031, but not for the medium problem, *r*(62) = −.042, *p* = .744. This indicates that, apart from the medium problem in the BG condition, numeracy was a significant predictor of judgments. However, despite the initial impression when viewing Fig. 4 that the pattern of correlations is somewhat different between presentation format conditions, the coefficients for each problem type in fact do not differ significantly. Furthermore, there is no interaction between numeracy and format for any of the three problems. Hence, the evidence here did not support our prediction: Numeracy generally explained a similar proportion of variance in contingency judgments in both conditions.^3^

## Discussion

Understanding covariation information in order to determine a potential causal relationship requires the consideration and integration of four pieces of information: the frequency with which the candidate cause and the effect co-occur, the frequency that the candidate cause is present and the effect does not occur, the frequency that the effect is present in the absence of the candidate cause, and the frequency with which the candidate cause and the effect are both absent. However, poor numerical ability and the use of heuristics can undermine the normative appreciation of that evidence. This experiment explored the degree to which presentation format and numerical ability had an impact on judgments of contingency in three covariation problems which ranged in level of difficulty. Results indicate that presentation format did influence contingency judgments, although the nature of the difference in judgments depended on the level of problem difficulty.

### The effects of presentation format

Consistent with the findings of Stone et al. (2003), when information was presented in a composite bar chart, judgments were more accurate—closer to the normatively correct value—for all three problems than when presented numerically in a contingency table. Participants viewing the contingency information in a summary table format were more inclined to (incorrectly) perceive a positive contingency for the hard and medium problems, and while they were able to detect the negative association for the easy problem, they tended to overestimate its magnitude. Interestingly then, while the largest effect was seen for the easy problem in terms of a reduction in the magnitude of the negative judgment, it is for the hard problem (and to a lesser extent for the medium problem) that the use of composite bar graphs had the largest impact by facilitating the shift from an intuitive, positive response to a normative, negative response.

These results suggest that a simple and nonoptimal judgment heuristic was cued when contingency information was presented numerically in a 2 × 2 matrix, whereby cells were weighted asymmetrically, as demonstrated by Crocker (1982), Shaklee and Tucker (1980), Smedslund (1963), and Wasserman et al. (1990) for example. Specifically, judgments appear to have been based on the contents of Cell A, or a comparison of Cells A and C, the frequency in Cell A being much greater than that in Cell C in the hard problem, only marginally greater in the medium problem, and smaller in the easy problem. This is consistent with studies which have found that people use nonnormative strategies in which focus is placed on these cells (Arkes & Harkness, 1983; McKenzie, 1994; Shaklee & Mims, 1981; Shaklee & Tucker, 1980; Ward & Jenkins, 1965), prefer to confirm a hypothesis by examining cases containing the property of interest (Klayman & Ha, 1987), and neglect denominators (Reyna & Brainerd, 2008). These results are also predicted by FTT whereby the contents of Cell A were encoded as a focal gist of the problem, and confusion was created by the overlapping classes. However, this pattern of judgments was eliminated for participants viewing information in the graphical format: They were more likely to correctly infer the negative association for the hard and medium problems, and in addition, the overestimation for the easy problem was attenuated relative to the contingency table condition. That the mean judgment for the easy problem in the BG condition was considerably more negative than the normative −.1 suggests that even when viewing this graphical format, some consideration was still given to the relative sizes of target classes—namely, the magnitudes of the number of plants that bloomed with and without the fertilizer. This was the only problem for which the latter was greater and hence represented by a larger bar area (see Fig. 2), potentially inflating the size of the negative judgment.

Thus, presenting covariation information in a composite bar graph resulted in more consistent contingency judgments regardless of difficulty. Furthermore, the use of a graphical format appears to have inhibited denominator neglect, as judgments were more normatively correct despite the unequally sized treatment groups. This supports research which has found that a graphical form that draws attention to both the numerator and denominator of a risk ratio, and their nested relationship, enhances comprehension (Ancker et al., 2006; Garcia-Retamero & Cokely, 2013, 2017; Garcia-Retamero et al., 2010; Stone et al., 2003).

### The effects of numeracy

Our results indicate that numeracy was associated with performance in the contingency judgment task for both presentation formats, and for all levels of difficulty; participants achieving a higher score on the numeracy scale tended to make judgments closer to the normatively correct value. We conjectured that low numeracy would have less impact on performance when information was viewed in a composite bar graph relative to a contingency table, due to the reduced requirement to manipulate numerical information. However, this was not the case. Although there were some differences in the pattern of correlations between numeracy and judgments in the two presentation formats, more noticeably for the medium problem, numeracy accounted for a similar proportion of variance in the two conditions. Thus, viewing contingency information in the graphical form enhanced performance for individuals both high and low in numeracy. This finding contrasts with that of Galesic et al. (2009), who found that graphical representations were especially useful in communicating medical risk to low-numeracy participants. However, Galesic and colleagues used icon arrays with equal base rates, meaning that the proportions represented were easier to compare in the graphical format than in the present experiment, which used unequal base rates. Developmental researchers have demonstrated some time ago the difficulties experienced by children in choosing which of two containers is more likely to result in a draw of the target object (e.g., a blue token from two containers with blue and red tokens) when absolute and relative frequencies of the target object are designed to conflict (see Reyna & Brainerd’s, 1994, review, and in particular Fig. 11.3).

### Implications, limitations, and future research

The results reported here provide additional support to the body of evidence which suggests that graphical representations can enhance comprehension of numerical information, and that problems in communication occur because inappropriate information formats are often used. Specifically, graphical formats that reduce detail and instead direct attention to the features of the problem that are necessary to form a correct solution, are most likely to be useful in achieving that solution (e.g., Liersch & McKenzie, 2009). For contingency judgments, the salient feature is the proportion (not the absolute number) of effect-occurring cases within each of the cause-present and cause-absent classes, which should be compared, and hence made evident in the display. When this is done, strategies that incorporate concepts of proportion are more easily employed. Thus, for the problems in this experiment, positive contingencies were often perceived in the CT condition because the difference in the number of effect-occurring cases looked larger than in the BG condition where the size of the more inclusive cause-present/cause-absent class could be seen more clearly.

We propose that the use of a composite bar chart could be effective in conveying health risk information and alleviate some of the issues identified with alternative graphical formats. For example, icon arrays can make risks appear less serious than a numerical representation as they show both people who are affected by a risk and those who are not (Galesic et al., 2009), thus drawing attention to what is often a much larger number of unaffected people, and discouraging a focus on just those affected (the numerator of the risk ratio), which has been posited as the reason behind greater seriousness of risks observed in some numerical and visual formats (Reyna & Brainerd, 1994; Stone et al., 2003). Galesic et al. (2009) also found that otherwise equivalent risks appear more serious when a larger overall number of icons is used, which fits with a ratio-bias effect (Reyna & Brainerd, 2008). Composite bar charts, on the other hand, while also effective in reducing denominator neglect and visually displaying the part-to-whole relationship (e.g., Stone et al., 2003), do so with reference to bar areas rather than individual icons, and hence may focus attention even further on proportions rather than the absolute numbers involved. However, where the desire is to emphasize risk (for example, to encourage behaviour change) or deemphasize it (for example, to alleviate false fears), this should also be considered when deciding on the most appropriate presentation format.

The participant sample in this experiment was perhaps unusual in terms of age, education, and numeracy. This may have affected the analysis of the effects of numeracy and makes comparisons with other studies more difficult. Future studies should recruit more educationally heterogeneous samples. Alternatively, a more challenging measure of numeracy should be employed that can adequately differentiate among higher performing individuals or improve discriminability among a general population (for suggestions, see Cokely, Galesic, Schulz, Ghazal, & Garcia-Retamero, 2012; Frederick, 2005; Weller et al., 2013).

The problems in the present experiment incorporated a marginal negative contingency and unequal base rates, making them particularly difficult. While this strengthens the case for composite bar charts as an effective means of communication of covariation information, further investigation is necessary to determine whether the advantage holds for a wider range of cause–effect contingencies (positive as well as negative). Furthermore, all three problems had identical normative solutions, but participants might have avoided giving the same response each time for pragmatic reasons. This should be investigated by varying contingency type and level as a within-subjects factor. As an anonymous reviewer pointed out, the lack of explicit marginals in the CT condition (which represent denominators and are therefore necessary for normative calculations) might have limited the comparison with responses in the BG condition in which the row totals could be inferred more directly by the overall length of each bar. However, we must note that the numbers involved in this experiment were relatively congenial—that is, amenable to simple additions and ratio calculations, since cell frequencies were multiples of 10 and for all three problems the frequencies for Cells C (120) and D (30) were constant; in turn, frequencies were not always exactly discernible in the BG condition (since the composite segments did not always align precisely with the gridlines). Still, a contingency-table-with-marginals condition would nevertheless be an interesting addition in future experiments. As suggested by another reviewer, an avenue for future research could investigate whether making proportions salient in a numerical format (e.g., using percentages) enhances performance in a similar way to a graphical format. It would also be interesting to determine the degree to which the outcome bias—that is, the influence of the overall base rate of the effect on contingency judgments—is moderated by presentation format as suggested by F. Vallée-Tourangeau et al. (2008), as well as numeracy.

## Electronic supplementary material

ESM 1 (DOCX 58 kb)

## Footnotes

1 In the absence of any directly comparable studies reporting effect sizes with which to conduct an a priori power analysis, our sample size was determined to allow the detection of a small effect of 0.3, assuming α = .05 and 1 − β = .50.

2 Mauchly’s test indicated that the assumption of sphericity was not met, χ^2^(2) = 13.96, *p* = .001.

3 As there was a nonsignificant difference between numeracy scores in the CT and BG conditions, and similar gradients for the regression slopes can be seen in Fig. 4, the effect of numeracy was investigated further by adding it as a covariate in the ANOVA. Homogeneity of regression slopes was confirmed, *F*(1, 123) = 0.06, *p* = .813. The analysis revealed a significant effect of numeracy on judgments, *F*(1, 124) = 18.79, *p* < .001, η_p_^2^ = .132, and the significant main effect for level of difficulty remained, *F*(1.81, 225.95) = 39.18, *p* < .001, η_p_^2^ = .239. After controlling for numeracy, the main effect of presentation format remained nonsignificant, *F* < 1, and the interaction between presentation format and level of difficulty remained significant, *F*(1.81, 223.80) = 8.38, *p* = .001, η_p_^2^ = .063. These results confirm the original analysis.

## References

[CR1] Allan LG (1980). A note on measurement of contingency between two binary variables in judgment tasks. Bulletin of the Psychonomic Society.

[CR2] Ancker JS, Senathirajah Y, Kukafka R, Starren JB (2006). Design features of graphs in health risk communication: A systematic review. Journal of the American Medical Informatics Association.

[CR3] Arkes HR, Harkness AR (1983). Estimates of contingency between two dichotomous variables. Journal of Experimental Psychology: General.

[CR4] Chapman GB, Liu J (2009). Numeracy, frequency, and Bayesian reasoning. Judgment and Decision Making.

[CR5] Cokely ET, Galesic M, Schulz E, Ghazal S, Garcia-Retamero R (2012). Measuring risk literacy: The Berlin Numeracy Test. Judgment and Decision Making.

[CR6] Cokely ET, Kelley CM (2009). Cognitive abilities and superior decision making under risk: A protocol analysis and process model evaluation. Judgment and Decision Making.

[CR7] Crocker J (1982). Biased questions in judgment of covariation studies. Personality and Social Psychology Bulletin.

[CR8] Fagerlin A, Wang C, Ubel PA (2005). Reducing the influence of anecdotal reasoning on people’s health care decisions: Is a picture worth a thousand statistics?. Medical Decision Making.

[CR9] Frederick S (2005). Cognitive reflection and decision making. Journal of Economic Perspectives.

[CR10] Galesic M, Garcia-Retamero R, Gigerenzer G (2009). Using icon arrays to communicate medical risks: Overcoming low numeracy. Health Psychology.

[CR11] Garcia-Retamero R, Cokely ET (2013). Communicating health risks with visual aids. Current Directions in Psychological Science.

[CR12] Garcia-Retamero R, Cokely ET (2017). Designing visual aids that promote risk literacy: A systematic review of health research and evidence-based design heuristics. Human Factors.

[CR13] Garcia-Retamero R, Galesic M, Gigerenzer G (2010). Do icon arrays help reduce denominator neglect?. Medical Decision Making.

[CR14] Gigerenzer G, Gaissmaier W, Kurz-Milcke E, Schwartz LM, Woloshin S (2007). Helping doctors and patients make sense of health statistics. Psychological Science in the Public Interest.

[CR15] Kao SF, Wasserman EA (1993). Assessment of an information integration account of contingency judgment with examination of subjective cell importance and method of information presentation. Journal of Experimental Psychology: Learning, Memory, and Cognition.

[CR16] Klayman J, Ha YW (1987). Confirmation, disconfirmation, and information in hypothesis testing. Psychological Review.

[CR17] Larkin JH, Simon HA (1987). Why a diagram is (sometimes) worth ten thousand words. Cognitive Science.

[CR18] Liersch MJ, McKenzie CR (2009). Duration neglect by numbers—And its elimination by graphs. Organizational Behavior and Human Decision Processes.

[CR19] Lipkus IM (2007). Numeric, verbal, and visual formats of conveying health risks: Suggested best practices and future recommendations. Medical Decision Making.

[CR20] Lipkus IM, Samsa G, Rimer BK (2001). General performance on a numeracy scale among highly educated samples. Medical Decision Making.

[CR21] McKenzie CR (1994). The accuracy of intuitive judgment strategies: Covariation assessment and Bayesian inference. Cognitive Psychology.

[CR22] Peters E (2012). Beyond comprehension: The role of numeracy in judgments and decisions. Current Directions in Psychological Science.

[CR23] Peters E, Dieckmann N, Dixon A, Hibbard JH, Mertz CK (2007). Less is more in presenting quality information to consumers. Medical Care Research and Review.

[CR24] Peters E, Hibbard J, Slovic P, Dieckmann N (2007). Numeracy skill and the communication, comprehension, and use of risk-benefit information. Health Affairs.

[CR25] Peters E, Västfjäll D, Slovic P, Mertz CK, Mazzocco K, Dickert S (2006). Numeracy and decision making. Psychological Science.

[CR26] Reyna VF (2004). How people make decisions that involve risk: A dual-processes approach. Current Directions in Psychological Science.

[CR27] Reyna VF, Brainerd CJ, Wright G, Ayton P (1994). The origins of probability judgment: A review of data and theories. *Subjective probability*.

[CR28] Reyna VF, Brainerd CJ (2007). The importance of mathematics in health and human judgment: Numeracy, risk communication, and medical decision making. Learning and Individual Differences.

[CR29] Reyna VF, Brainerd CJ (2008). Numeracy, ratio bias, and denominator neglect in judgments of risk and probability. Learning and Individual Differences.

[CR30] Reyna VF, Nelson WL, Han PK, Dieckmann NF (2009). How numeracy influences risk comprehension and medical decision making. Psychological Bulletin.

[CR31] Schirillo JA, Stone ER (2005). The greater ability of graphical versus numerical displays to increase risk avoidance involves a common mechanism. Risk Analysis.

[CR32] Schwartz LM, Woloshin S, Black WC, Welch HG (1997). The role of numeracy in understanding the benefit of screening mammography. Annals of Internal Medicine.

[CR33] Shaklee H, Mims M (1981). Development of rule use in judgments of covariation between events. Child Development.

[CR34] Shaklee H, Tucker D (1980). A rule analysis of judgments of covariation between events. Memory & Cognition.

[CR35] Shaklee H, Wasserman EA (1986). Judging interevent contingencies: Being right for the wrong reasons. Bulletin of the Psychonomic Society.

[CR36] Sirota M, Juanchich M (2011). Role of numeracy and cognitive reflection in Bayesian reasoning with natural frequencies. Studia Psychologica.

[CR37] Smedslund J (1963). The concept of correlation in adults. Scandinavian Journal of Psychology.

[CR38] Stone ER, Sieck WR, Bull BE, Yates JF, Parks SC, Rush CJ (2003). Foreground: Background salience: Explaining the effects of graphical displays on risk avoidance. Organizational Behavior and Human Decision Processes.

[CR39] Stone ER, Yates JF, Parker AM (1997). Effects of numerical and graphical displays on professed risk-taking behavior. Journal of Experimental Psychology: Applied.

[CR40] Vallée-Tourangeau F, Payton T, Murphy RA (2008). The impact of presentation format on causal inferences. European Journal of Cognitive Psychology.

[CR41] Vallée-Tourangeau G, Abadie M, Vallée-Tourangeau F (2015). Interactivity fosters Bayesian reasoning without instruction. Journal of Experimental Psychology: General.

[CR42] Ward WC, Jenkins HM (1965). The display of information and the judgment of contingency. Canadian Journal of Psychology.

[CR43] Wason PC (1960). On the failure to eliminate hypotheses in a conceptual task. Quarterly Journal of Experimental Psychology.

[CR44] Wasserman EA, Dorner WW, Kao SF (1990). Contributions of specific cell information to judgments of interevent contingency. Journal of Experimental Psychology: Learning, Memory, and Cognition.

[CR45] Weller JA, Dieckmann NF, Tusler M, Mertz CK, Burns WJ, Peters E (2013). Development and testing of an abbreviated numeracy scale: A Rasch analysis approach. Journal of Behavioral Decision Making.

[CR46] Wolfe CR, Fisher CR, Reyna VF (2013). Semantic coherence and inconsistency in estimating conditional probabilities. Journal of Behavioral Decision Making.

[CR47] Wolfe CR, Fisher CR, Reyna VF, Hu X (2012). Improving internal consistency in conditional probability estimation with an intelligent tutoring system and web-based tutorials. International Journal of Internet Science.

[CR48] Wu CM, Meder B, Filimon F, Nelson JD (2017). Asking better questions: How presentation formats influence information search. Journal of Experimental Psychology: Learning, Memory, and Cognition.

[CR49] Zikmund-Fisher BJ, Ubel PA, Smith DM, Derry HA, McClure JB, Stark A, Fagerlin A (2008). Communicating side effect risks in a tamoxifen prophylaxis decision aid: The debiasing influence of pictographs. Patient Education and Counseling.

