# Supplementary material for: The effects of numeracy and presentation format on judgments of contingency
Source: Mem Cognit. 2020 Aug 26;49(2):389–99. doi: 10.3758/s13421-020-01084-8 (PMC7886725; doi:10.3758/s13421-020-01084-8)
Supplement: Supplementary file 1 — (DOCX 58 kb) [file 13421_2020_1084_MOESM1_ESM.docx]

**Supplementary Materials**

**Numeracy Scale**

(from Lipkus, Samsa, & Rimer, 2001)

Imagine that you rolled a fair, six-sided die 1,000 times.

Out of the 1,000 rolls, how many times do you think the die would come up even (2, 4, or 6)?

In the BIG WIN LOTTERY, the chances of winning a £10,000 prize is 1%.

What is your best guess about how many people would win a £10,000 prize if 1,000 people each buy a single ticket for the BIG WIN LOTTERY?

In the ACME PUBLISHING SWEEPSTAKES, the chance of winning a car is 1 in 1,000.

What percentage of tickets to ACME PUBLISHING SWEEPSTAKES win a car?

Which of the following numbers represents the biggest risk of getting a disease?

1 in 100 1 in 1000 1 in 10

Which of the following numbers represents the biggest risk of getting a disease?

1% 10% 5%

If Person A’s risk of getting a certain disease is 1% over ten years, and person B’s risk is double that of A’s, what is B’s risk?

If Person A’s risk of getting a certain disease is 1 in 100 over ten years, and person B’s risk is double that of A’s, what is B’s risk?

The chance of getting a certain disease is 10%.

Out of 100 people, how many would be expected to get the disease?

The chance of getting a certain disease is 10%.

Out of 1000 people, how many would be expected to get the disease?

The chance of getting a certain disease is 20 out of 100.

This would be the same as having a ____% chance of getting the disease.

The chance of getting a viral infection is 0.0005.

Out of 10,000 people, about how many of them are expected to get infected?

**Fictitious Scenario for Contingency Judgment Task**

(adapted from Kao & Wasserman, 1993)

Kingston Flowering Plants Laboratory and the Lanyu
Imagine you are employed by Kingston Flowering Plants Laboratory which has developed 3 experimental fertilizers (labelled F1, F2 and F3) for promoting the Lanyu (an exotic plant from Brazil) to bloom. Each fertilizer has been tested on a completely different group of plants. Within each group, some plants were given the fertilizer and some were not. All plants were kept in the same conditions and were watered at the same time daily. Over a period of one month, the plants were monitored closely to determine which ones bloomed and which did not, and the results recorded. Your task is to look at these results and evaluate the effectiveness of each fertilizer.

Given the results for each fertilizer, you must rate on a scale from -10 to +10 its effectiveness in promoting the Lanyu to bloom. A score of -10 means that you think the fertilizer has a strong negative effect on the plant’s blooming; a score of 0 means that you think the fertilizer has no effect on the plant’s blooming; and a score of +10 means that you think the fertilizer has a strong positive effect on the plant’s blooming. Of course, you can use any score in between these values on the scale.

On the next 3 screens you will see the results for each of the 3 fertilizers. The results for F1, F2 and F3 will appear in no particular order.

**Example Contingency Judgment Problem in the CT Condition**

**Fertilizer F1**
210 plants were given the fertilizer and bloomed.
90 plants were given the fertilizer but did not bloom.
120 plants did not receive the fertilizer but bloomed.
30 plants did not receive the fertilizer and did not bloom.

These results can be summarized in the following table:


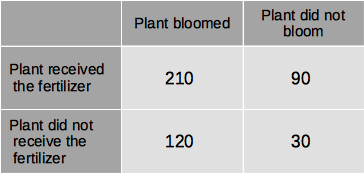


How effective do you think fertilizer F1 was in promoting the Lanyu to bloom?

**Example Contingency Judgment Problem in the BG Condition**

**Fertilizer F1**
210 plants were given the fertilizer and bloomed.
90 plants were given the fertilizer but did not bloom.
120 plants did not receive the fertilizer but bloomed.
30 plants did not receive the fertilizer and did not bloom.

These results can be summarized in the following bar chart:


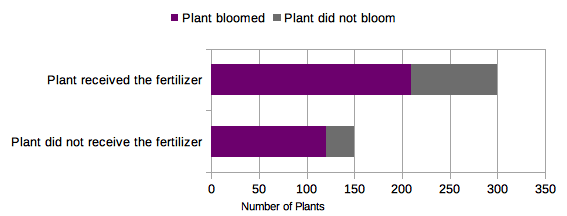


How effective do you think fertilizer F1 was in promoting the Lanyu to bloom?
